# Ethnic variations in orthodontic treatment need in London schoolchildren

**DOI:** 10.1186/1472-6831-5-8

**Published:** 2005-09-27

**Authors:** Mhd Nour Alkhatib, Raman Bedi, Claire Foster, Pooja Jopanputra, Sue Allan

**Affiliations:** 1 Department of Dental Public Health, Guy's King's & St Thomas' Dental Institute, Floor 2, Caldecot Road, Denmark Hill Campus, London SE5 9RW, UK; 2 WHO Collaborating Centre for Research, Education and Service in oral health, disability and culture, UK; 3 North West London Community Dental Services, Hammersmith and Fulham Primary Care Trust. Parsons Green Centre, 5-7 Parsons Green, London. SW6 4UL, UK

## Abstract

**Background:**

The study was carried out to determine the prevalence of orthodontic treatment need in children from minority ethnic groups and compare the need to the white population. The second objective was to explore variations in agreement between subjective and objective treatment need in a multiethnic context using the aesthetic component of Orthodontic Treatment Need Index (IOTN AC).

**Methods:**

A cross-sectional study in North West London, 14 schools were randomly selected from the 27 schools in the two boroughs of Harrow and Hillingdon. Comparison between objective and subjective treatment need was carried out using IOTN AC index. Clinical orthodontic treatment need was also recorded using the dental health component of Orthodontic Treatment Need Index (IOTN DHC).

**Results:**

2,788 children were examined and completed the questionnaire. 16% of the study population were already wearing appliances or had finished orthodontic treatment. Of the remaining children; 15% had definite need for treatment using the dental health component of the IOTN. There was no significant variation in the need for orthodontic treatment between different ethnic backgrounds (*P *> 0.05) whether using the AC or DHC components of the IOTN index. However, poor agreement was detected between professional and subjective assessment of ethnic minority of orthodontic treatment need using IOTN AC index.

**Conclusion:**

Orthodontic treatment need in children of ethnic minorities does not differ significantly from the vast majority of white children. However treatment need based on aesthetic index continues to vary in all ethnic groups from the professional aesthetic assessment

## Background

Orthodontic treatment is one of the most costly and challenging issues to NHS dentistry. In 2003, fees for orthodontic treatment accounted for more than a quarter of all child fees [1]. Whilst the focus has been often on strategies to improve access and increase budget of orthodontic care, evaluation of the criteria used for defining the need has received little attention. Most orthodontic indices have been primarily based on white populations; this may raise a concern since the demography of the United Kingdom has changed rapidly in the last few decades. The majority of ethnic minorities reside in the England region with London being the most diversified city; almost 30% of London inhabitants are of ethnic minorities [2].

The health needs of ethnic minorities may be different from the white population. In a comparative review of subgingival calculus formation, association between plaque formation and ethnicity has been reported [3]. These needs may be particularly different when they are related to aesthetics, where confounding factors such as cultural or societal values play a major role in shaping these needs. It has been shown that black people are more likely to have different orthodontic needs than other ethnicities; they are more likely to have class III occlusion, anterior open bites and mid-line diastema than their white or Asian counterparts [4,5]. Kiyak [6] showed that Pacific Asians had different dental beliefs and behaviours and different views about aesthetics compared to Caucasians.

Two of the properties of the ideal orthodontic index laid out by Shaw et al [7] were; 1- sensitive to the needs of the patients, 2- acceptable to both the public and the profession. Orthodontic care in the UK is currently provided on the definite need basis of the Index of Orthodontic Treatment Need (IOTN) devised by Brook and Shaw [8]. Under this category only severe cases are eligible for treatment under the scheme. IOTN, however, has two components; the Dental Health Component (DHC) and the Aesthetic Component (AC). The latter has gained increased popularity in recent years because a) the primary motive for seeking orthodontic treatment is improving appearance [9,10] and an aesthetic component would seem necessary to any diagnostic tool. The aesthetic component should therefore be accounted for when planning treatment using the DHC component, b) since patient satisfaction is the one of the main outcomes of treatment, a tool should be devised to be used by both patients and professionals to measure this outcome; the AC component has been shown to be capable of facilitating this task [10]. Moreover, a strong correlation between the AC component and psychosocial outcomes was reported by Bennett et al [11]. Mandall et al [12] also reported an association between child self esteem and IOTN AC but not with IOTN DHC. However, the AC is more subjective and less reliable than the DHC; studies which compared the need based on the two components demonstrated poor correlation [13-15]. Researchers suggested adjustment to the DHC defined need in order to balance for the aesthetic component.

The aims of the study were, first to explore the need for treatment in a multiethnic community. Secondly to assess whether the need for orthodontic treatment in ethnic minorities differs from the white population based on the dental health component and on the aesthetic component and, thirdly to test the agreement between normative and perceived need for orthodontic care across all ethnicities.

## Methods

The study took place 2002/2003. All schools in the boroughs of Harrow and Hillingdon were included in the initial sampling. Fourteen out of the twenty seven schools in the two boroughs were selected using a one to one simple sampling technique. The required sample size for each ethnic group was based on our pilot study [14]. Children aged 12 to14 were included, the study population consisted of 3,500 children. Dental examination was carried out in accordance with BASCD criteria [16], DMFT was recorded together with IOTN DHC and AC, the examiner (PJ) was calibrated for both DMFT and IOTN indexes with high intra examiner reliability (for the Aesthetic Component of IOTN, the examiner achieved a weighted kappa score of 0.82 showing very good agreement, sensitivity of 100% and specificity of 84.2%). The questionnaire was developed by the Transcultural Oral Health Centre at an earlier stage [14]. This was then modified and tested in a second pilot.

Measurement of IOTN AC was recorded using a 10-point analogue scale with two pictures at each end of the scale representing IOTN AC scores of 1 and 10, this was used to avoid the bias reported by Burden and Pine [17] in recording children's perception of the IOTN AC. Normative IOTN AC and DHC were recorded according to the calibration criteria. Major ethnic groups in the UK described by the national census were used to identify the ethic background of the child. Ethnicity was therefore categorized as follows:

- White: English, Irish or any other Caucasian white.

- Black: African, Caribbean or black other.

- Asian: Indian, Sri Lankan, Bangladeshi and Pakistani.

- Chinese: Mainland Chinese, Korean, Taiwanese and Japanese.

- Mixed: Any mixed race.

Data was encoded and entered onto SPSS software, descriptive analysis was undertaken to report frequency distributions of IOTN scores. Logistic regression was undertaken to explore the ethnic variation with agreement/disagreement with different IOTN AC threshold levels. Kappa coefficient test was utilized to estimate the agreement between normative and subjective need for treatment.

Ethical approvals were obtained from the local ethic committee in the two boroughs. Consent letters were sent out to schools and parents.

## Results

Out of the 3,500 children, 2,788 children were examined and completed the questionnaire representing a response rate of 80%. Distribution of sex was almost equal (48% males Vs 52% females). Over half of children (54%) were of white ethnic background. 12% Blacks, 25% Indians, 4% Chinese and 6% were of mixed race.

442 children (16%) were undergoing orthodontic treatment during the screening, when recording IOTN AC all children were included to explore the need based on aesthetic grounds irrespective of previous or current orthodontic treatment. However when DHC was used, only those who had not received or undergoing any orthodontic treatment were included in the analysis.

To ensure reliability of assessing normative need, 280 subjects of the 2,788 were re-examined. The Cohen's Kappa score would give an indication of the level of agreement between the first and second reading thus indicating intra-examiner reliability.

Kappa scores can either be measured from a 2 × 2 table, giving a simple Kappa score. Alternatively, it can be weighted to account for 'near miss' scores. Both methods were used to measure intra-examiner reliability. The simple Kappa score was 0.89 representing a very good agreement.

Using weighted kappa score, the same principle is used as for measuring simple Kappa but instead the weight is taken into account, Microsoft Excel^® ^was used to calculate weighted Kappa values. For intra-examiner agreement in this study, weighted Kappa was found to be 0.79, representing substantial agreement.

### Perceived need

**Figure 1:**
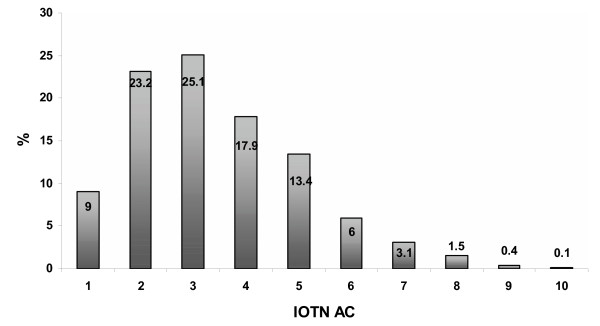
Children's assessment of IOTN AC.

Using the IOTN AC index children graded their teeth accordingly. Almost half of children (48%) scored their teeth as 2 or 3 on the index. Less than 2% had severe scores (8–10). Distribution of responses to self-grading of teeth is illustrated graphically in figure 1. Grouping the responses in three categories to estimate treatment need based on the IOTN AC index revealed that three quarters (75%) of the children perceived no need for treatment (AC = 1–3), whilst 23% perceived borderline need (AC = 4–7) and only 2% perceived definite need for treatment (AC = 8–10). Distribution of responses according to ethnicity is presented in table 1 where children from black ethnic minority had the least perceived need for treatment compared to other ethnicities. Variations were small in the definite need for treatment except for children from Chinese ethnic background who did not report any definite need for treatment.

**Table 1:** Children's rated IOTN AC by treatment category and ethnicity

**Treatment need**	**IOTN AC score**	**White**	**Black**	**Indian**	**Chinese**	**Mixed (others)**
**No need for treatment**	1–4	1123 (74.9)	263 (80.2)	493 (72.1)	86 (74.8)	135 (83.3)
**Moderate/Borderline need**	5–7	338 (22.5)	59 (18.0)	179 (26.2)	29 (25.2)	24 (14.8)
**Need for treatment**	8–10	38 (2.5)	6 (1.8)	12 (1.8)		3 (1.9)
**Total**		1499 (100.0)	328 (100.0)	684 (100.0)	115 (100.0)	162 (100.0)

### Normative need using IOTN AC

**Figure 2:**
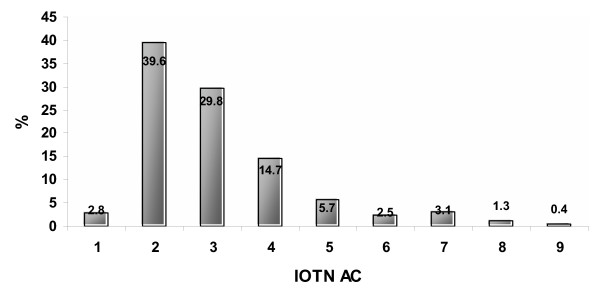
Dentist's assessment of IOTN AC.

The examiner scored over two thirds (69%) of children's teeth as 2 or 3 on the IOTN AC index. No child was assessed as having grade 10 and less than 2% had grade 8 and 9. Results are illustrated in figure 2. Based on treatment need categories 87% of children were assessed as having no need for treatment, 11% had borderline need for treatment and 2% has definite need for treatment need. The main descriptive difference is clear at the 'no' and borderline need levels for treatment.

Differences between ethnic groups were less obvious in the professional assessment; the majority of children from all ethnic backgrounds were assessed as having low need for treatment (AC 1–3). Results are summarised in table 2.

**Table 2:** Examiner's rated IOTN AC by treatment category and ethnicity

**Treatment need**	**IOTN AC score**	**White**	**Black**	**Indian**	**Chinese**	**Mixed (others)**
**No need for treatment**	1–4	1293 (86.3)	292 (89.0)	590 (86.3)	103 (89.6)	146 (90.1)
**Moderate/Borderline need**	5–7	177 (11.8)	33 (10.1)	77 (11.3)	12 (10.4)	16 (9.9)
**Need for treatment**	8–10	29 (1.9)	3 (0.9)	17 (2.5)		
**Total**		1499 (100.0)	328 (100.0)	684 (100.0)	115 (100.0)	162 (100.0)

### Normative need using DHC component

Using the dental health component, two thirds of children (68%) had no need for treatment, 17% had moderate need for treatment and 15% had definite need for treatment. Variations in need for treatment with ethnicity are presented in table 3. Children from black ethnicity had less need for treatment than did their white peers, whereas children of Chinese and Indian ethnicities had slightly more need for treatment. However these differences were not statistically significant when entered in a regression model using ethnicity as an explanatory variable.

**Table 3:** Normative need (IOTN DHC) and ethnicity

**Treatment need**	**IOTN DHC**	**White**	**Black**	**Indian**	**Chinese**	**Mixed (others)**
**No need for treatment**	1–2	878 (69.0)	213 (72.9)	365 (65.2)	54 (62.1)	94 (69.6)
**Moderate/Borderline need**	3	208 (16.4)	40 (18.6)	104 (18.6)	19 (21.8)	25 (18.5)
**Need for treatment**	4–5	186 (14.6)	39 (13.4)	91 (16.3)	14 (16.1)	16 (11.9)
**Total**		1272 (100.0)	292 (100.0)	560 (100.0)	87 (100.0)	135 (100.0)

### Agreement between normative and perceived treatment need

**Figure 3:**
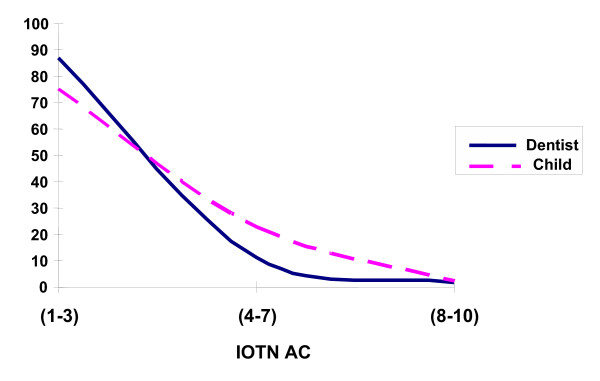
Comparison between children's and dentist's ratings of IOTN AC.

Differences between children's and professionals' AC scores are illustrated in figure 3 which is a combination of figure 1 and 2. Variations are clear at the borderline level of need (AC = 4–7). In the logistic regression using the need definition of the scale as a cut-off point for comparison, the influence of ethnicity was not statistically significant in the perceived assessment of IOTN AC neither in normative assessment IOTN DHC (*P *> 0.05). Agreement between children's ratings and the dentist's rating is presented in table 4, at the low need level (AC 1–4) there was agreement in 80% of cases. This decreased to 50% at the borderline need level (AC 5–7). At the definite need level the number of cases was small so was the agreement (6%). Since the majority of responses of both children and the dentist are in the low need and borderline need level, the scale mid point was used (AC 5) to test the agreement/disagreement across different ethnicities. Cohen Kappa's test revealed poor overall agreement (K = 0.18). In the ethnic context; white ethnicity demonstrated a kappa score of 0.15, black, Indian, Chinese and mixed scored 0.27, 0.18, 0.43, and 0.48 respectively indicating poor agreement with the professional assessment.

**Table 4:** Agreement between the dentist and children on IOTN AC

*	**Low (1–4)**	**Moderate (5–7)**	**High (8–10)**	**Total**
**Low (1–4)**	1938 (80.0%)	147	15	2100
**Moderate (5–7)**	442	156 (49.5%)	31	629
**High (8–10)**	44	12	3 (6.1%)	59
**Total**	2424	315	49	2788

*Dentist's scores are displayed vertically in the table

## Discussion

In this cross-sectional study, the proportion of children who were professionally assessed as having a clinically definite need for treatment is lower than previously reported in the child dental health survey for the UK [18]. The perceived need based on the AC component was very low and the overall rating of AC varied slightly between children of ethnic minorities. The only small difference was seen in black children where they perceived their teeth as more attractive than did their white counterparts but again these differences were not statistically significant. Ahmed et al [14] reported that children from black ethnic minorities were more likely to perceive less need for treatment compared to professional assessment. In contrast, Otuyemi et al [19] showed no difference in the perceptions of dental aesthetics between adult Nigerians and Americans.

In this study ethnicity did not influence orthodontic need for treatment based on clinical or aesthetic grounds. However children of Indian and Chinese ethnicities had a slightly higher clinical need for treatment. These results are mirrored in Mandall et al [15] study where they reported that ethnicity did not influence self grading of aesthetics and that Asian adolescents had more need for orthodontic treatment compared to Caucasians using the DHC component.

The disagreement between professional and children's grading of the AC is not unexpected, however this disagreement was not influenced by ethnicity, a result in line with and confirming findings from Mandall et al study [15].

## Conclusion

Perception and prevalence of malocclusion in children of ethnic minorities is not different from the White ones. Perhaps their perceptions may have been influenced by the cultural and societal circumstances in their current place of living and these may be different from the perceptions held by peers living in their original countries.

Self-perception of aesthetics of malocclusion differed significantly from professional assessment; however this disagreement was not confined to ethnicity. The majority of disagreements between children and dentists were at the borderline level of need, perhaps a different threshold of need definition may resolve this discrepancy. It should be noted that disagreement is also influenced by the prevalence of malocclusion and previous experience, in this study the prevalence of severe malocclusion based on aesthetic grounds was low therefore overall agreement should be interpreted carefully.

## Competing interests

The author(s) declare that they have no competing interests.

## Authors' contributions

MNK managed the study and analysed the data. RB is the principal investigator he wrote the paper with MNK. CF, PJ and SA coordinated with the schools and local health authorities, collected the data and contributed to writing of the discussion.

## Pre-publication history

The pre-publication history for this paper can be accessed here:



## References

[B1] (2004). Dental Review 2003–2004.

[B2] (2003). Census 2001 National Report for England and Wales.

[B3] Roberts-Harry EA, Clerehugh V (2000). Subgingival calculus: where are we now? A comparative review. J Dent.

[B4] Trottman A, Elsbach HG (1996). Comparison Of Malocclusion In Pre-school Black And White Children. Am J Orthod.

[B5] Brunelle JA, Bhat M, Lipton JA (1996). Prevalence and distribution of selected occlusal characteristics in the US population, 1988–1991. J Dent Res.

[B6] Kiyak HA (1981). Dental beliefs, behaviors and health status among Pacific Asians Caucasians. Community Dent Oral Epidemiol.

[B7] Shaw WC, O'Brien KD, Richmond S, Brook P (1991). Quality Control In Orthodontics: Risk/Benefit Considerations. Br Dent J.

[B8] Brook PH, Shaw WC (1989). The Development of an index of orthodontic treatment priority. Eur J Orthod.

[B9] Gochman DS (1975). The measurement and development of dentally relevant motives. J Public Health Dent.

[B10] Jacobson A (1984). Psychological aspects of dentofacial esthetics and orthognathic surgery. Angle Orthod.

[B11] Bennett ME, Tulloch JF, Vig KW, Phillips CL (2001). Measuring orthodontic treatment satisfaction: questionnaire development and preliminary validation. J Public Health Dent.

[B12] Mandall NA, Wright J, Conboy FM, O'Brien KD (2001). The relationship between normative orthodontic treatment need and measures of consumer perception. Community Dent Health.

[B13] Birkeland K, Boe OE, Wisth PJ (1996). Orthodontic concern among 11-year-old children and their parents compared with orthodontic treatment need assessed by index of orthodontic treatment need. Am J Orthodont.

[B14] Ahmed B, Gilthorpe MS, Bedi R (2001). Agreement between normative and perceived orthodontic need amongst deprived multiethnic school children in London. Clin Orthod Res.

[B15] Mandall NA, McCord JF, Blinkhorn AS, Worthington HV, O'Brien KD (2000). Perceived aesthetic impact of malocclusion and oral self-perceptions in 14–15-year-old Asian and Caucasian children in greater Manchester. Eur J Orthod.

[B16] Pitts(1) NB, Evans(2) DJ, Pine(3) C (1997). British Association for the Study of Community Dentistry (BASCD) Diagnostic Criteria for Caries Prevalence Surveys – 1996/97. Community Dent Health.

[B17] Burden DJ, Pine CM (1995). Self-perception of malocclusion among adolescents. Community Dent Health.

[B18] O'Brien M (1994). Children's dental health in the United Kingdom 1993.

[B19] Otuyemi OD, Ogunyinka A, Dosumu O, Cons NC, Jenny J, Kohout FJ, Jakobsen J (1998). Perceptions of dental aesthetics in the United States and Nigeria. Community Dent Oral Epidemiol.

